# Cloud Based AI-Driven Video Analytics (CAVs) in Laparoscopic Surgery: A Step Closer to a Virtual Portfolio

**DOI:** 10.7759/cureus.29087

**Published:** 2022-09-12

**Authors:** Ahmed Gendia

**Affiliations:** 1 General and Colorectal Surgery, Northmapton General Hospital, Northampton, GBR

**Keywords:** laparoscpic surgery, medical education & training, surgical videos, ai and machine learning, cloud computing

## Abstract

Aims: To outline the use of cloud-based artificial intelligence (AI)-driven video analytics (CAVs) in minimally invasive surgery and to propose their potential as a virtual portfolio for trainee and established surgeons.

Methods: An independent online demonstration was requested from three platforms, namely Theator (Palo Alto, California, USA), Touch Surgery™ (Medtronic, London, England, UK), and C-SATS® (Seattle, Washington, USA). The assessed domains were online and app-based accessibility, the ability for timely trainee feedback, and AI integration for operation-specific steps and critical views.

Results: The CAVs enable users to record surgeries with the advantage of limitless video storage through clouding and smart integration into theatre settings. This can be used to view surgeries and review trainee videos through a medium of communication and sharing with the ability to provide feedback. Theator and C-SATS® provide their users with surgical skills scoring systems with customizable options that can be used to provide structured feedback to trainees. Additionally, AI plays an important role in all three platforms by providing time-based analysis of steps and highlighting critical milestones.

Conclusion: Cloud-based AI-driven video analytics is an emerging new technology that enables users to store, analyze, and review videos. This technology has the potential to improve training, governance, and standardization procedures. Moreover, with the future adaptation of the technology, CAVs can be integrated into the trainees’ portfolios as part of their virtual curriculum. This can enable a structured assessment of a surgeon’s progression and degree of experience throughout their surgical career.

## Introduction

Intraoperative video recording is increasingly becoming an essential part of modern-day surgical practice and serves multiple uses, including structured surgical training, continued performance assessment, and quality improvement [1]. With advances in surgical innovations, the recording of surgeries has become more accessible, mainly due to the introduction of cloud-based storage [2].

Cloud computing comprises a system of storing, sharing, and accessing data that is regulated and controlled by a group of remote servers. This technology allows centralized data storage and easy online access through multiple devices or connections [3]. For decades, cloud computing has been leveraged by big data companies to process and store seemingly large amounts of data for commercial use [4]. With the recent advances in healthcare technologies, clinical institutes are adopting the use of cloud-based data storage [5]. In surgical practice, cloud-based applications that are aided by artificial intelligence (AI) and computer vision have been developed to record, store, and analyze surgical videos

Therefore, cloud technology has facilitated the recording and storage of surgeries through secure servers, but without the limitation of outdated or conventional methods of storage [2]. This ability allows the introduction of a huge amount of data into AI for analysis that is based on different parameters, such as segmentation of surgeries, instrumentation, key performance analysis, and anatomical landmarks. These features have introduced the possibility of structured feedback to surgeons and have helped to improve safety and critical skill development [6].

With the increased application and adaptation of cloud-based AI-driven platforms in surgery, several platforms have become available. This study aims to provide insight into some of the established applications and to propose the use of these applications to create a virtual portfolio or video library for surgeons.

## Materials and methods

An independent online demonstration was requested of 11 platforms that were identified by online searches through multiple search engines. The inclusion criteria were that the platforms must be fully supported cloud platforms with AI-integration solutions.

Exclusion criteria included platforms that are not publicly available to users, or still under development, or with limited access to surgical specialties. Also, platforms with no AI integration or clouding were excluded.

Invitations to different platforms were sent during September 2021 with a one-month notice to showcase the platforms. A demonstration was requested from these platforms to include a live walkthrough and trial of the features and abilities of the platform with an interview to provide information about the technology.

The assessed domains were smart integration into the theater setting, which represents the feasibility of their services in the operating theater, and these include online and app-based accessibility, governance compliance, removal of sensitive information, feedback, and quantifiable scoring systems, sharing content and live connectivity, and AI-integration services.

## Results

Demonstrations were provided by Theator (Palo Alto, California, USA), Touch Surgery™ (Medtronic, London, England, UK), and C-SATS® (Seattle, Washington, USA). To varying degrees, all three platforms can provide safe, secure video cloud storage with integrated AI and computer vision. Nonetheless, each platform provides different services.

**Figure 1:**
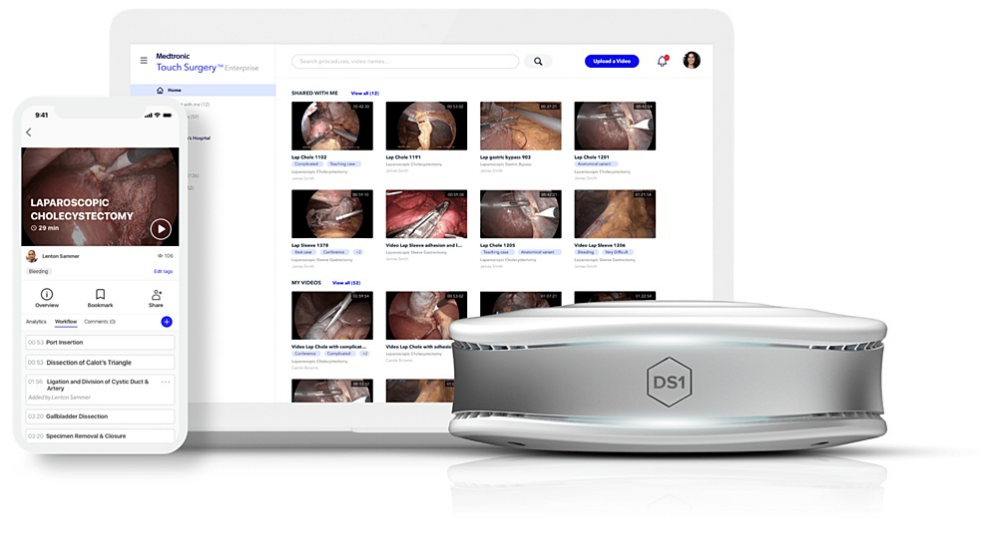
The Touch Surgery™ interphase Left foreground: The smartphone view showing case review with surgical phase analysis, Centre background: Webpage displaying a surgical video library, Right foreground: The DS1 smart computer for the operating room with built-in AI anonymization capabilities that enables secure and easy access to surgical videos. All rights reserved and permission to utilize the figure is obtained from Medtronic, ©2022.

**Figure 2:**
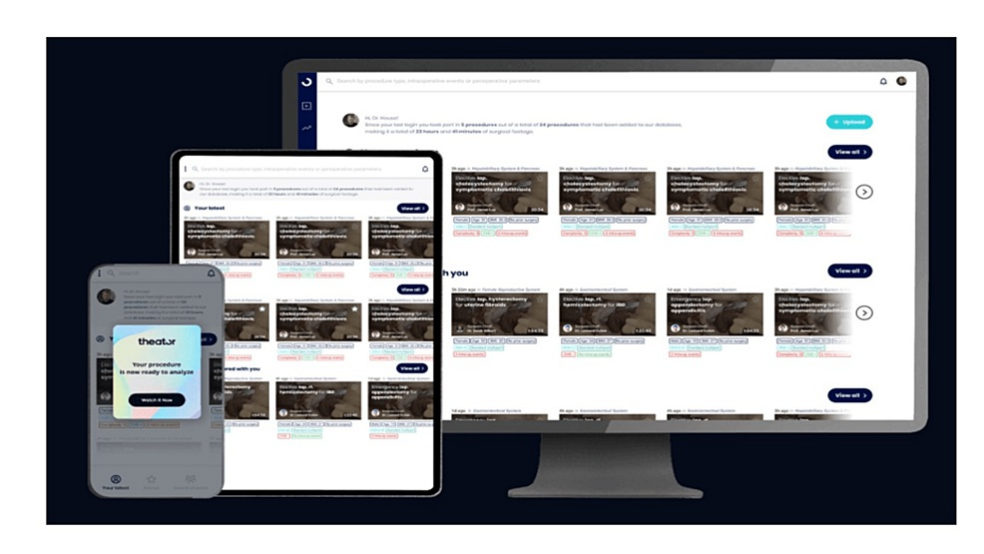
The different interphases of Theator All rights reserved and permission to utilize the figure is obtained from Theator, ©2022.

First, the platforms enable smart integration into the laparoscopic or robotic setting through a smart device (tablet or phone) and integration into the laparoscopic or robotic sets, which are connected to the cloud servers. This feature enables instant uploading and recording of videos to the platform and eases the effort of transferring recorded videos for the stack and uploading them to the secure platform. Furthermore, the applications ensure that the surgeon has the ability to select the type of surgery as well as the primary operator and the assistant (Figure 1). Second, the web and mobile applications interphase allows surgeons to utilize applications through different interphases and devices. This provides the surgeons with easy access to their personalized portfolio of uploaded surgeries as well as the ability to view other surgeons’ procedures (Figure 2). For governance compliance in their respective regions, these three platforms provide compliance and security approval with regard to healthcare and patient safety information safety standards such as the Health Insurance Portability and Accountability Act (HIPAA), International Organization for Standardization (ISO), American Institute of Certified Public Accountants (AICPA), General Data Protection Regulation (GDPR), Health Information Technology for Economic and Clinical Health (HITECH) and Service Organization Control 2 (SOC2), which are enhanced by the removal of sensitive information i.e., patient details or out-of-body video, a key feature in all platforms and is accomplished with the help of AI.

**Figure 3:**
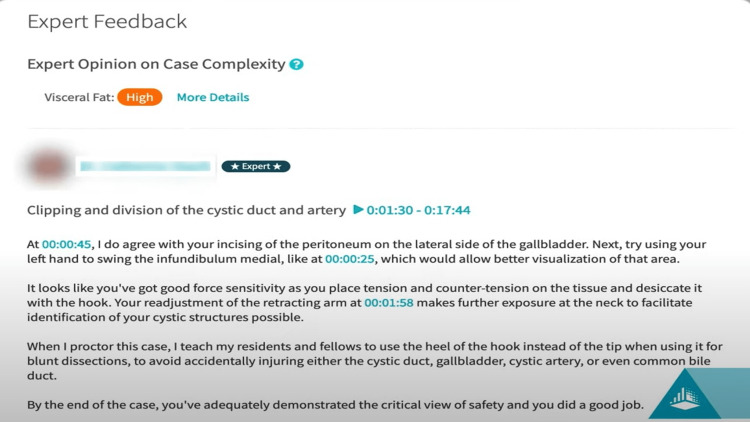
Example of feedback on a case provided by a member of the C-SATS® expert panel All rights reserved and permission to utilize the figure is obtained from C-SATS®, ©2022.

Another important feature is feedback and quantifiable skill-scoring systems. The ability to provide feedback on surgical proficiency is considered an active feature in the platforms, which allows users and trainers to either comment or provide structured feedback for colleagues or trainees (Figure 3). Additionally, skill scoring systems such as Global Evaluative Assessment of Robotic Skills (GEARS) and Global Operative Assessment of Laparoscopic Skills (GOALS) are used, and customizable scoring systems constitute a powerful added feature.

The ability to share content and live connectivity on all platforms allows users to share their videos with other users, local surgical groups, and platform-driven groups. Moreover, live connectivity allows users to stream live surgeries remotely with delegated users, which makes the platforms a powerful communication tool among surgeons.

**Figure 4:**
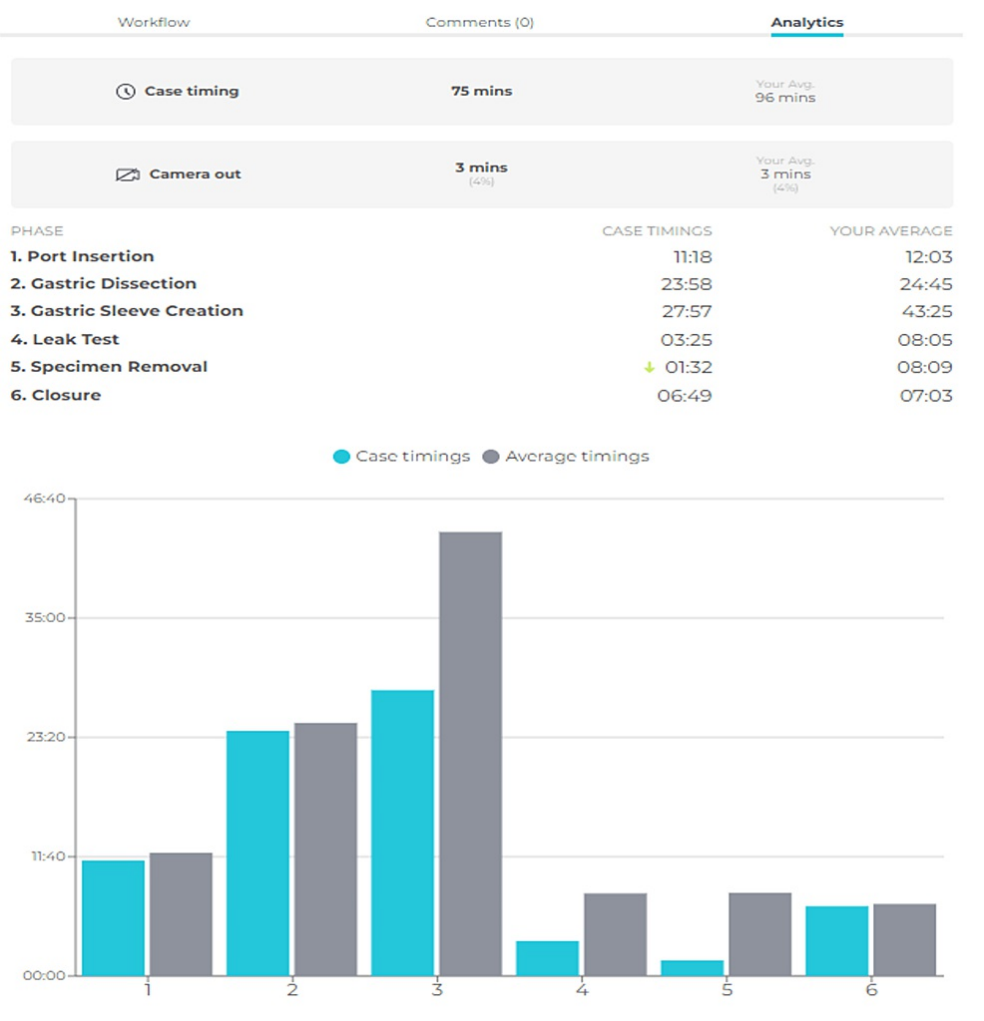
A portfolio of analytics provided by Touch Surgery™ to help surgeons identify new ways to advance techniques All rights reserved and permission to utilize the figure is obtained from Medtronic, ©2022.

**Figure 5:**
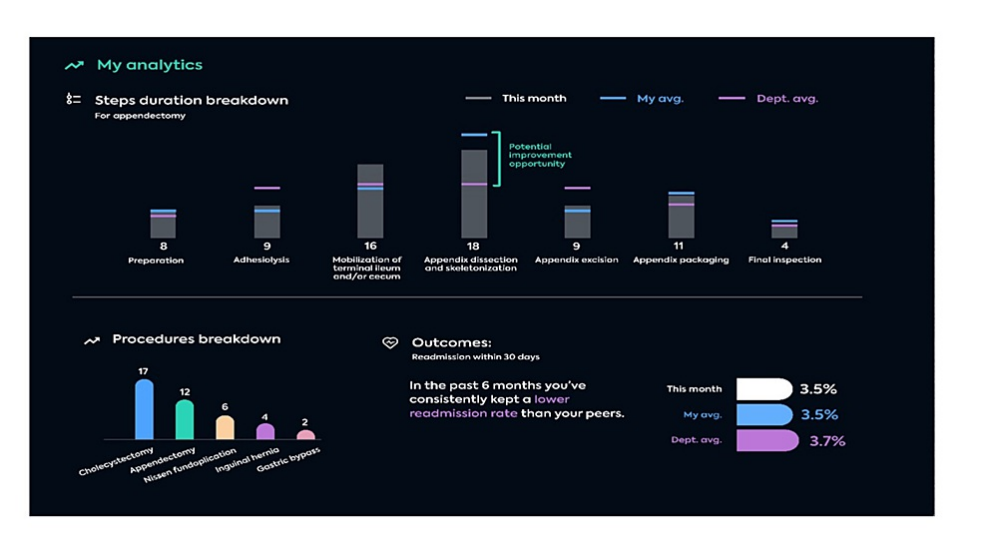
Example of different analytics highlighted and analyzed by Theator All rights reserved and permission to utilize the figure is obtained from Theator, ©2022.

Additionally, AI integration provides a powerful tool for surgeons to annotate and analyze their surgeries. Although different features are found in each platform, the following points summarize some of the key features that include the removal of sensitive patient information and the annotation and segmentation of surgical steps, which helps segment surgical videos into steps to facilitate the highlighting of different stages of surgery. This feature enables video review in an easy and sequential manner (Figure 4). Annotation of events and safety milestones help highlight certain events, such as a critical view of safety in laparoscopic cholecystectomy as well as bleeding or bowel spillage events (Figure 5). Finally, the time analytics feature provides analytics based on the time spent on each step and gives the surgeon a report that can be compared to their overall average time of performing the procedure or of different steps (Figure 4).

## Discussion

Video data collection in endoscopic surgery has significant potential and applications to improve surgical proficiency by ensuring increased compliance with standard techniques and the reduction of inter-surgeon variations [7,8]. Moreover, video recording can be a useful teaching tool for surgical training as it provides a wide source of educational materials to help in developing skills and improving the safety of procedures [9]. In addition, the recording of surgeries can play a role in a patient's education and provide clarity to their interventional procedures [10]. 

Currently, surgical videos are either recorded or stored for a limited duration on the laparoscopic storage stack and are subsequently discarded. Conventional video storage is limited by cost and the facility’s access to quality local storage systems. These systems can lead to missing or damaged data.

Cloud-based AI-driven video analytics (CAVs) is a novel technology that facilitates the recording of surgical videos and their storage in a limitless cloud environment. This ability ensures the integrity of saved data through secure cloud servers and affords the advantage of AI-assisted removal of sensitive information. Furthermore, AI plays a role in CAVs by enabling the analysis of videos according to different parameters, including segmentation of surgical steps, annotation of events and safety milestones, and facilitating analytics based on timing. All of these features make these platforms a powerful tool for surgeons.

Manual video analysis and annotations require significant time and effort to process videos that are highly accurate and adhere to a standard systematic approach [11]. Machine learning (ML) analysis of videos with the help of AI has provided a useful and accurate tool for segmenting and analyzing surgical videos [12], which provides a more efficient and informative way of reviewing surgical videos [13]. Machine learning can enable the analysis of surgical videos based on segmentation or phase recognition [14], which provides a time stamp to the surgery based on different procedural steps [15]. Another feature of AI-based analysis is related to different identification of instruments and anatomical landmarks [6].

The analysis of surgeries through these platforms can generate a report for surgeons which helps generate insights into which steps in the procedure can be improved. However, the variability and complexity of the surgery can play a role in time stamping and in assessing the quality of the performance [16]. Although some of the platforms have an additive difficulty score based on the surgeon’s input, developing a machine-based classification will help to universally quantify the surgery according to its difficulty [17].

Another aspect is the quantitative assessment of surgical skills in ML that has been utilized to quantify laparoscopic or robotic surgical skills through different scores. For example, Objective Structured Assessment of Technical Skills (OSATS) and GEARS have been used to quantify surgeons’ performance based on kinetics and tissue handling. These tools, or rubrics, can help to determine a surgeon's proficiency level [18,19]. Similarly, the available applications provide a range of quantitative skills assessments, whether AI-driven or performed by humans. Moreover, with the advances in algorithms to process huge amounts of data, a full AI-based assessment will be available and can be utilized to assess and provide feedback to surgeons. This functionality might help achieve global and universal competency schemes to deliver standardized and safer patient-centered services.

Although surgical videos recording and cloud-based storage are becoming powerful tools for various purposes, concerns have hampered implementations of video recording and sharing. Data protection and privacy are considered major issues concerning the new technology. Mainly, the processing of patient information and videos can raise many legal issues [7]. However, appropriate patient consent and regulation according to the regions or health institutes are to be followed and applied when using this technology. Although the platforms are designed to ensure anonymity and security with the power of AI, all global and regional formalities for cloud storage should be implemented.

Another challenge is cloud data security, as cloud-based data are liable to have security issues mainly such as data thefts, cyber-attacks, and possible damage to the data. Cloud providers usually store their data in multiple data centers that are located in different geographical regions to ensure high-speed collection and recall of massive data. However, in case of disasters or cyber-attacks, those cloud databases could be vulnerable [20]. The current applications are indeed anonymous when it comes to a patient’s information. Also, with global investments and protected databases, those issues could be minimized.

With the advances in healthcare technologies and the implementation of technologies in surgical training, the need for the development of a virtual portfolio or video library for surgeons is becoming increasingly essential. These platforms provide surgeons with up-to-date video analytics of their performance and enable continuous monitoring. This feature could be used for training purposes, as well as interviews and assessments, rather than the reliance on conventional methods of surgical logbooks and competency forms.

Regarding the limitation of reviewing the platforms in this article, the demonstrations were provided based on requests, and the interviews with the platform's creators do not reflect the daily usage of the platforms. And so, this article can show some of the advantages and disadvantages only briefly. Nevertheless, an overview of the importance of video analytics has been demonstrated as they are a newly emerging technology in surgical practice, which can evolve with time to provide both surgeon and patient with services that can enhance their experience.

## Conclusions

With the advances and wide applications of AI and cloud-driven applications in surgery, CAVs will provide surgeons i.e., both trainees and experts, with powerful tools which can analyze, view, and provide critical feedback to improve surgical practice and governance while ensuring quality and standardized measures. Therefore, the need to develop a virtual portfolio with the help of CAVs would be an essential milestone in surgical training and development not only for trainees but also for established surgeons.

Additionally, these applications can provide a monitoring tool for clinical governance rather than conventional operative notes. This will require further integration of CAVs in everyday surgical practice to ensure clarity and safety for both surgeons and patients.
